# Evaluation of Psoriasis Genetic Risk Based on Five Susceptibility Markers in a Population from Northern Poland

**DOI:** 10.1371/journal.pone.0163185

**Published:** 2016-09-22

**Authors:** Marta Stawczyk-Macieja, Krzysztof Rębała, Aneta Szczerkowska-Dobosz, Joanna Wysocka, Lidia Cybulska, Ewa Kapińska, Agnieszka Haraś, Paulina Miniszewska, Roman Nowicki

**Affiliations:** 1 Department of Dermatology, Venereology and Allergology, Medical University of Gdańsk, Gdańsk, Poland; 2 Department of Forensic Medicine, Medical University of Gdańsk, Gdańsk, Poland; University of Alabama at Birmingham, UNITED STATES

## Abstract

Psoriasis genetic background depends on polygenic and multifactorial mode of inheritance. As in other complex disorders, the estimation of the disease risk based on individual genetic variants is impossible. For this reason, recent investigations have been focused on combinations of known psoriasis susceptibility markers in order to improve the disease risk evaluation. Our aim was to compare psoriasis genetic risk score (GRS) for five susceptibility loci involved in the immunological response (*HLA-C*, *ERAP1*, *ZAP70*) and in the skin barrier function (*LCE3*, *CSTA*) between patients with chronic plaque psoriasis (n = 148) and the control group (n = 146). A significantly higher number of predisposing alleles was observed in patients with psoriasis in comparison to healthy individuals (6.1 vs. 5.2, respectively; P = 8.8×10^−7^). The statistical significance was even more profound when GRS weighted by logarithm odds ratios was evaluated (P = 9.9×10^−14^). Our results demonstrate the developed panel of five susceptibility loci to be more efficient in predicting psoriasis risk in the Polish population and to possess higher sensitivity and specificity for the disease than any of the markers analyzed separately, including the most informative HLA-C*06 allele.

## Introduction

Psoriasis is a chronic, inflammatory skin disease. It affects approximately 2–4% of Caucasian population, with higher prevalence in northern Europe [1–3].

The role of genetic background in the pathogenesis of psoriasis is undoubted. Up to date, it has been classified as a complex disease with multifactorial mode of inheritance. The interactions between particular genes as well as genetic and environmental factors play an important role in the induction of psoriatic process.

According to the results of various linkage analyses performed on large family cohorts, *psoriasis susceptibility 1* (PSORS1) *locus* on chromosome 6 was proved to be significantly associated with psoriasis [4–6]. Amongst 10 genes identified in the *locus*, HLA-C*06 is considered to be the main psoriasis susceptibility marker [7–13]. Independent large-scale molecular studies confirmed a strong association of this allele with early-onset type of the disease in Caucasians [8,10,11,13]. Nevertheless, HLA-C*06 is estimated to be responsible for less than 50% of genetic predisposition to psoriasis [4–6]. This fact suggests presence of non-MHC susceptibility polymorphisms, that may explain the complex nature of the disease.

Recent genome-wide association studies (GWAS) have led to identification of more than forty novel genetic risk factors associated with psoriasis. They include different polymorphisms of genes involved in the skin barrier function (*LCE3B*, *LCE3C*, *CSTA*), interleukin 12/interleukin 23 and NF-kappaB dependent signaling pathways (*IL12B*, *IL23A*, *IL23R*, *TRAF3IP2*, *TYK2*, *TNFAIP3*, *TNIP1*, *NFKBIA*, *REL*, *IFIH1*, *IL28RA*) as well as immunological response mediated by CD8+ lymphocytes (*ERAP1*, *ZAP70*) [14,15]. Although proteins encoded by these genetic markers show biological epistasis, the majority of known polymorphisms associate with the risk of psoriasis independently. Compared to HLA-C*06 alone, this effect provides only scant explanation of complexity in genetics of psoriasis.

In case of polygenic disorders, single genetic markers have small impact on phenotype and their presence do not allow to establish a definitive diagnosis. In order to improve disease risk prediction, certain combinations of such markers have been recently proposed [16–20].

In this study, we compared psoriasis genetic risk score (GRS) between patients with psoriasis and healthy controls by analysis of five known susceptibility markers: HLA-C*06, *LCE3C_LCE3B-del* and three single nucleotide polymorphisms (SNPs): rs26653 located within the *ERAP1* gene, rs17695937 linked to the *ZAP70* gene and rs17589 located within the *CSTA* gene. This is the first report on GRS estimation for a panel of psoriasis susceptibility loci in a population of patients with psoriasis from Eastern Europe.

## Materials and Methods

### Selection of genetic risk markers

In our study, we focused on psoriasis susceptibility genes involved in the immunological response (*HLA-C*, *ERAP1*, *ZAP70*) and in the skin barrier function (*LCE3*, *CSTA*). For two genes, alleles with well documented genetic correlation with psoriasis: HLA-C*06 and *LCE3C_LCE3B-del*, were chosen. The selection of SNPs from other psoriasis susceptibility loci (*CSTA*, *ERAP1* and *ZAP70*) was based on results of previous GWAS and large cohort studies. The markers included into the study demonstrated the strongest correlation with the risk of psoriasis in European populations and comprised c.162 T>C within the *CSTA* gene (as studied by Vasilopoulos *et al*. [21] and identified in our study as rs17589), rs26653 within the *ERAP1* gene and rs17695937 linked to the *ZAP70* gene (Table 1).

**Table 1:** The review of SNPs of *CSTA*, *ERAP1* and *ZAP70* genes, which were tested for correlation with the risk of psoriasis.

Gene	SNP	Reference	OR (95% CI)	P value
*CSTA*	CSTA SNP 1	Samuelsson et al. [22]	N/A	1.00
	CSTA SNP 3		N/A	1.00
	g.-190 T>C	Vasilopoulos et al. [21]	0.768 (0.45, 1.31)	0.298
	c.162 T>C*		3.45 (2.28, 5.22)	<0.001
	c.344 C>T		1.505 (0.97, 2.34)	<0.054
*ERAP1*	rs26653*	Lysell et al. [23]	1.31 (1.16–1.48)	0.00006
	rs30187		1.16 (1.03–1.30)	0.02
	rs27524		1.10 (0.98–1.23)	0.11
	rs27044	Tang et al. [24]	0.86 (0.83–0.89)	2.16×10^−14^
	rs26653*		0.87 (0.84–0.91)	5.27×10^−12^
	rs27524	Yang et al. [25]	1.27 (1.10–1.46)	1.17×10^−3^
	rs151823	Sun et al. [26]	0.89 (0.85–0.92)	9.32×10^−9^
	rs27524	Oostveen et al. [27]	1.55 (1.18 to 2.03)	0.002
	rs27524	Strange et al. [28]	1.27 (1.18–1.38)	4.24×10^−11^
*ZAP70*	rs17695937*	Strange et al. [28]	N/A	2.37×10^−7^

*SNPs selected for our study

SNP: single nucleotide polymorphism; N/A: not available

### Subjects

A total of 294 individuals from northern Poland were enrolled in the case-control study, including 148 patients with psoriasis (79 males and 69 females) and 146 unrelated healthy controls (83 males and 63 females; Table 2). The mean age of the case group was 43.3 years (range: 18–83). The mean age of the controls was 42.0 years (range: 19–84). No statistically significant differences in the gender distribution (Fisher exact test: P = 0.56) and in the age of the enrolled individuals (U Mann-Whitney test: P = 0.37) were revealed between the two groups. The patients were recruited at the Department of Dermatology, Venereology and Allergology at the University Clinical Centre in Gdańsk. They were qualified into the study based on the presence of skin lesions characteristic for plaque psoriasis assessed during dermatological examination. Guttate psoriasis, pustular psoriasis or psoriatic erythroderma were clinical types excluded from the study. There were neither personal nor familial history of psoriasis and/or psoriatic arthritis in the control group. All participants provided written informed consent and the study was approved by ethical committee of the Medical University of Gdańsk (NKBBN/181/2012).

**Table 2:** Cohort characteristics.

**Patients with psoriasis**		**Men**	**Women**	**Total**
	**n**	79	69	148
	**Mean age (years)**	41.50	42.70	43.30
**Control group**		**Men**	**Women**	**Total**
	**n**	83	63	146
	**Mean age (years)**	43.30	43.30	42.00

### Genotype analysis

DNA samples were extracted from peripheral blood samples, using a modified method by Lahiri and Nurnberger [29]. HLA-C*06 genotypes were determined by the optimized three-step procedure. Polymerase chain reaction (PCR) with sequence-specific primers (PCR-SSP) was used for specific detection of HLA-C*06. PCR with analysis of restriction fragment length polymorphism was used to distinguish between HLA-C*06 homozygous and heterozygous subjects. Homozygous genotypes were additionally analyzed for nonspecific digestion by PCR-SSP [30,31]. Identification of *LCE3C_LCE3B-del* alleles was performed by PCR with evaluation of amplified fragment length polymorphism (PCR-AFLP), described previously in the study by de Cid *et al*. [32]. Three different primers were used for the reaction (S1 Table). A specific product of 240 base pairs (bp) in length without the deletion was amplified with LCE3C_F and LCE3C_R primers. *LCE3C_LCE3B-del* allele of 199 bp in length was detected by LCE3C_F and LCE3CR2D primers. After optimization of temperature of hybridization of specific primers used to detect SNPs of *ERAP1*, *CSTA* and *ZAP70* genes, discrimination of rs26653, rs17589 and rs17695937 alleles was performed by PCR-SSP method (S1 Table). In order to ensure quality control in all cases, a negative non-template control as well as an internal positive control of amplification were used to eliminate genotype mistakes. The PCR products were analyzed by polyacrylamide gel electrophoresis and stained with silver.

### Genetic risk score calculation

As far as some DNA samples did not show products of amplification of one or more markers, the GRS evaluation based on five susceptibility loci was performed on the population of 126 patients with psoriasis and 100 healthy controls with complete genotypes for five genetic polymorphisms. MedCalc 16 (MedCalc Software bvba) was used for multivariate logistic regression analysis adjusted for gender and age and for odds ratio (OR) estimation. Two methods described in the study by Chen *et al*. were used to obtain GRS scores [19]. The count GRS (cGRS) calculation was based on a simple counting of alleles predisposing to psoriasis, whereas weighted GRS (wGRS) was computed as the number of risk alleles multiplied by logarithm odds ratio (Log(OR)) for each of five alleles in the panel. Descriptive statistics of cGRS and wGRS values were performed with the use of STATISTICA 12 package (StatSoft Inc.). Since both parameters did not display normal distribution, the nonparametric U Mann-Whitney test was used in order to compare cGRS and wGRS values in the case-control cohort. Receiver operating characteristic (ROC) curves for the five individual susceptibility markers as well as for cGRS and wGRS values were obtained in STATISTICA 12. Areas under ROC curves (AUCs) for different predictors were compared by a method described by DeLong *et al*., as applied in MedCalc 16 software [33]. All statistical tests were two-sided and a standard significance level of 0.05 was used.

## Results

### The association of risk alleles with psoriasis

All allelic frequencies of patients and control subjects were in Hardy-Weinberg equilibrium (P>0.05). Out of the five tested psoriasis susceptibility markers, only HLA-C*06 and rs26653 G alleles showed statistically significant association with the disease (Table 3). The odds ratios for each of the predisposing alleles and corresponding confidence intervals are shown in Table 3.

**Table 3:** The odds ratios for each of the analyzed psoriasis genetic markers in the case-control samples from northern Poland.

Gene	Predisposing allele	OR	95% CI
*HLA-C*	HLA-C*06	7.42	3.94–13.98
*ERAP1*	rs26653 G	2.26	1.44–3.54
*LCE3*	*LCE3C_LCE3B-del*	1.19	0.75–1.89
*CSTA*	rs17589 T	1.12	0.54–2.32
*ZAP70*	rs17695937 A	1.05	0.59–1.87

OR: odds ratio; CI: confidence interval

### Genetic risk score calculation

The calculation of GRS was based on five risk alleles contributing to the risk of psoriasis regardless of statistical significance. Mean cGRS and wGRS in the population of patients with psoriasis were 6.1 and 3.2, respectively. The control group showed mean cGRS of 5.2 and mean wGRS of 1.9.The results revealed a significant difference between patients with psoriasis and healthy controls. Psoriatic subjects demonstrated significantly higher cGRS and wGRS values in comparison with healthy individuals (Table 4).

**Table 4:** Comparison of cGRS and wGRS values for the tested panel of five susceptibility markers between patients with psoriasis and healthy controls.

GRS	Group	Mean	SD	Median	IQR	P value*
cGRS	Psoriasis	6.06	1.19	6.00	2.00	8.76×10^−7^
	Control	5.21	1.25	5.00	2.00	
wGRS	Psoriasis	3.17	1.11	3.35	1.94	9.89×10^−14^
	Control	1.93	1.07	1.99	1.05	

*U Mann-Whitney test

**Fig 1:**
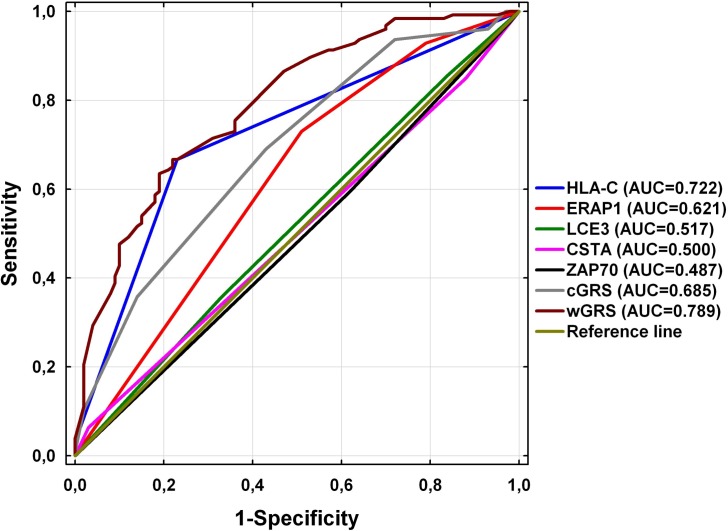
Comparison of ROC curves for prediction of psoriasis with the use of five psoriasis susceptibility markers analyzed separately and for cGRS and wGRS values obtained for these markers.

The ROC curves for prediction of psoriasis with the use of the tested markers are shown in Fig 1. The area under ROC curve (AUC) for wGRS was statistically larger than AUC for cGRS (0.789 vs. 0.685; P = 0.0002). It was also statistically larger than AUC for any of the five susceptibility markers analyzed separately, including the most informative HLA-C*06 allele (AUC = 0.722; P < 0.0001). No statistically significant difference was observed between AUCs for cGRS and HLA-C*06 (P = 0.3437).

## Discussion

Psoriasis is a common, inflammatory and hyperproliferative disease of skin and joints, with proven genetic basis. Numerous genome-wide association studies have recently led to identification of novel genetic risk markers associated with the disease [14,15]. Although their biological role may explain particular mechanisms arranged in the pathogenesis of psoriasis, the real effect of these variants on phenotype is modest. Due to complex nature of psoriasis, the disease risk prediction based on individual known susceptibility genes with low penetrance is limited. It has been shown that combining multiple risk loci with modest effects, identified previously in GWAS, into a global genetic risk score might improve identification of persons who are at increased risk for common complex diseases. This observation led to evaluation of genetic risk score based on the combination of known risk alleles in prediction of psoriasis [19].

Our aim was to determine the psoriasis GRS for five genetic risk variants whose contribution to the development of psoriasis was confirmed previously in linkage analysis and GWAS in large cohorts [7–13,23,24,28,34–36].

The association of *LCE3C_LCE3B-del* with the risk of psoriasis has been proved in ethnically diverse populations [34–36]. Numerous SNPs within the *ERAP1* gene have been analyzed in the context of predisposition to psoriasis, but the strongest association with the disease in a European population has been proved for rs26653 (P = 0.00006) [23] and was confirmed by Tang *et al*. in Chinese patients (P = 5.27×10^−12^) [24]. Up to date, different polymorphisms within the *ZAP70* gene have been suggested to predispose to psoriasis [28,37] and rs17695937 has been shown by Strange *et al*. to associate with the disease (P = 2.37×10^−7^) [28]. In 2008, Vasilopoulos *et al*. investigated three genetic markers in the *CSTA* gene, but the strongest effect on psoriasis risk was found for rs17589 (P<0.001) [21].

The odds ratios revealed that HLA-C*06, rs26653 G, *LCE3C_LCE3B-del*, rs17589 T and rs17695937 A alleles predispose to psoriasis, but the results were statistically significant only in case of HLA-C*06 and rs26653 G. The lack of significant association of *LCE3C_LCE3B-del*, rs17589 and rs17695937 alleles with psoriasis may result from the fact that the studied population was relatively small when compared to other investigations. Therefore, further analysis with a larger sample size is necessary to confirm these associations in Polish patients with psoriasis.

Independent studies revealed that weighted GRS is a better risk predictor in complex disorders than count GRS, as far as it accommodates differences in allelic influence on genetic predisposition to the disease [19,38]. In the present study, both mean wGRS and cGRS values were significantly higher in patients with psoriasis than in controls. The comparison of cGRS between the studied groups has shown that patients with psoriasis carry significantly more risk alleles than healthy individuals (on the average 6.1 alleles in psoriasis versus 5.2 alleles in the control group; P = 0.0000009). As mentioned above, cGRS calculation does not take into account the differences between particular alleles in predisposition to psoriasis, so our cGRS values are likely to be overestimated due to inclusion of predisposing alleles without statistical significance. Therefore, we additionally assessed wGRS values, and since they are weighted by logarithm odds ratios, the impact of alleles with OR values slightly exceeding 1 on the final wGRS values was negligible but still worthy of consideration as far as they may show statistical significance in larger cohorts as demonstrated by other authors. We found higher wGRS values in subjects with psoriasis than in healthy individuals (on the average 3.2 and 1.9 in patients with psoriasis and in the control group, respectively; P = 0.0000000000001). Thus, the observed difference was even more significant than in the cGRS evaluation. Moreover, also the comparison of ROC curves confirmed that wGRS assessed for the five markers is better for prediction of the risk of psoriasis than cGRS. The computed odds ratios revealed that within five genetic polymorphisms evaluated in this study, HLA-C*06 demonstrated the strongest effect on the risk of psoriasis. These findings are consistent with other large-scale observations and confirm HLA-C*06 to be a major psoriasis susceptibility genetic marker [7–13,19]. Anyway, our results show the developed panel of five susceptibility loci to be more efficient in predicting psoriasis risk and to possess higher sensitivity and specificity for the disease than any of the markers analyzed separately, including the most informative HLA-C*06 allele.

In 2011, Chen *et al*. worked out a panel of ten known psoriasis susceptibility genes, including *HLA-C* [19]. The study assumed evaluation of GRS and relationship between selected alleles and psoriatic subphenotypes. The results of the study showed that wGRS which included ten known risk alleles presented stronger association with psoriasis than any single SNP individually. Subjects in the highest wGRS quartile demonstrated a greater than 10-fold increased predisposition to the disease in comparison to individuals in the lowest quartile. Moreover, out of ten analyzed polymorphisms, the strongest signal was found at the *HLA-C* locus, which was in accordance with our results. The study also revealed that the prognostic value of nine genetic markers combined together was comparable to HLA-C*06 alone. The combination of nine non-MHC genetic markers presented similar association with the risk of psoriasis to *HLA-C*. Nevertheless, the cumulative effect was stronger in context of wGRS estimation than in the individual approach. On the other hand, none of nine risk alleles mentioned above was shown to interact with HLA-C*06. Our preliminary data did not show any interaction between analyzed genetic variants as well. On the contrary, other studies showed that polymorphisms in *LCE3C_LCE3B*, *ERAP1* and *ZAP70* genes, selected for our investigation, interact with HLA-C*06 [28,32,39–41]. Another study by Vasilopoulos *et al*. revealed a greater than 100-fold increase of psoriasis risk in patients carrying only three susceptibility markers: *HLA-C*, *CSTA* and D1S2346. In this case, investigated genetic markers also demonstrated epistatic interaction [20].

Genetic risk prediction based on panels comprising known risk alleles is a novel trend in context of complex disorders. The results of our study confirm that the combination of HLA-C*06, rs26653 G, *LCE3C_LCE3B-del*, rs17589 T and rs17695937 G displays a very significant effect on the risk of psoriasis and may be used in practice for its prediction. Further investigations with larger sample sizes from various ethnic groups are likely to confirm association of the studied markers with psoriasis in populations from Eastern Europe as well as to verify usefulness of the proposed panel of markers for prediction of psoriasis risk in other populations.

## Supporting Information

S1 Table Sequences of primers used for genotyping of *ERAP1*, *ZAP70* and *CSTA* variants. (DOCX)
