# Supplementary material for: Evaluation of Psoriasis Genetic Risk Based on Five Susceptibility Markers in a Population from Northern Poland
Source: PLoS One. 2016 Sep 22;11(9):e0163185. doi: 10.1371/journal.pone.0163185 (PMC5033405; doi:10.1371/journal.pone.0163185)
Supplement: S1 Table — (DOCX) [file pone.0163185.s001.docx]

**Supporting Information**

**S1 Table.** Sequences of primers used for genotyping of *ERAP1, ZAP70* and *CSTA* variants.

| Primer name | Primer sequence |
| --- | --- |
| ERAP1-FC | 5’GCAGTGCAATTTGCTCCTAAC3’ |
| ERAP1-FG | 5’GCAGTGCAATTTGCTCCTAAG3’ |
| ERAP1-R | 5’TGGGGAACCACGAAAGTAG3’ |
| ZAP70-FA | 5’CCTTCGGGGAGATATTTTTCA3’ |
| ZAP70-FG | 5’CTTCGGGGAGATATTTTCCG3’ |
| ZAP70-R | 5’GCTGGACGCTCATTTCTT3’ |
| CSTA-FC | 5’GAAGAAAAAACAAATGAGACTGAC3’ |
| CSTA-FT | 5’GAAGAAAAAACAAATGAGACTGAT3’ |
| CSTA-R | 5’TGAGAGTCCACCACTTG3’ |
